# The association between breastfeeding and prevalence of metabolic syndrome in women with a previous major pregnancy complication

**DOI:** 10.3389/fgwh.2026.1625603

**Published:** 2026-02-24

**Authors:** Maleesa M. Pathirana, Emily Aldridge, Prabha H. Andraweera, Melanie R. Wittwer, Katie Lowe, Susan Sierp, Gustaaf A. Dekker, Margaret A. Arstall

**Affiliations:** 1 School of Medicine, Adelaide University, Adelaide, SA, Australia; 2 Robinson Research Institute, Adelaide University, Adelaide, SA, Australia; 3 Department of Cardiology, Lyell McEwin Hospital, Elizabeth Vale, SA, Australia; 4 Division of Women’s Health, Lyell McEwin Hospital, Elizabeth Vale, SA, Australia

**Keywords:** breastfeeding, metabolic syndrome, preeclampsia, pregnancy complications, womens health, gestational diabetes, preterm delivery

## Abstract

**Introduction:**

Maternal complications of pregnancy, including preeclampsia, gestational diabetes mellitus, spontaneous preterm birth and placental abruption are individually associated with an increased risk of premature heart disease. Breastfeeding may reduce early postpartum cardiometabolic risk in this group. This study aimed the association between breastfeeding and the prevalence of cardiovascular risk factors and metabolic syndrome (MetS) in the early postpartum period amongst women with previous pregnancy complication.

**Methods:**

We conducted a cross-sectional analysis of 524 women who attended an appointment in a postpartum cardiovascular disease prevention clinic for women with previous pregnancy complications (HREC: TQEH/16/LMH/258). Breastfeeding status was self-reported and cardiovascular disease risk factors were assessed at approximately 6 months postpartum. MetS was reported as a marker of cardiovascular health, defined using the Harmonising The Metabolic Syndrome definition. Statistical analysis was reported using SPSS Version 27.

**Results:**

Breastfeeding for >5.5 months postpartum was associated with a lower prevalence of abnormal lipids (based on triglyceride and High Density Lipoprotein cut-offs for MetS), lower prevalence of treated hypertension and systolic blood pressure >130 mmHg. Women who breastfed for >5.5 months were 53% less likely to have MetS at 6 months postpartum (aOR 0.47 95%, CI 0.29–0.77). The prevalence of breastfeeding for >5.5 months was higher for women with previous gestational diabetes mellitus (GDM) (GDM 55% vs. non-GDM 44%, *p* = 0.025), and the prevalence of breastfeeding for >5.5 months was lower for women with a previous hypertensive disorder of pregnancy (HDP) (HDP 41% vs. non-HDP 59%, *p* < 0.001).

**Conclusion:**

Women who breastfeed for longer is associated with lower prevalence of MetS and its components in the early postpartum period. However, further studies are required to assess this association based on lactation intensity.

## 1 Introduction

Major pregnancy complications, such as gestational diabetes mellitus (GDM), hypertensive disorders of pregnancy (HDP), birth of a small for gestational age (SGA) infant, spontaneous preterm birth (sPTB) and placental abruption are all associated with at least a 2-fold increased risk of coronary artery disease later in life (1, 2). Clinical guidelines now recommend that women who experience these pregnancy complications should have early management of their cardiovascular risk through optimisation of risk factors and lifestyle intervention (3, 4). Postpartum cardiovascular clinics are being established to initiate primary prevention in this group of women with previous pregnancy complications, however such clinics are not yet routinely integrated into care and there is no consensus on how best to initiate prevention strategies for this unique group (5, 6).

Breastfeeding has been shown to reduce the long-term development of diabetes and heart disease in a general population (7–9). The *reset hypothesis* states that the deranged metabolic state during pregnancy is reversed more quickly with lactation (10). Therefore, encouraging breastfeeding in a cohort of women with previous pregnancy complications is likely to have positive effects on their future metabolic health. However, majority of the studies that assess the association between breastfeeding and cardiovascular health assess outcomes in women of middle and older age and do not account for pregnancy complications or early postpartum cardiometabolic health. Therefore, it is difficult to determine the direct effect that lactation has on long-term CVD outcomes without understanding how it is impacting metabolic health following pregnancy.

Women with these pregnancy complications have a shorter breastfeeding duration than those without uncomplicated pregnancy, due to pre-existing cardiometabolic risk factors and intrinsic factors during labour and birth in context of a high risk pregnancy (e.g., increased risk of caesarean section and infant separation inhibiting successful attachment and latch) (11, 12). This group more than likely over represents the proportion of cohorts that have low breastfeeding self-efficacy. Therefore, it is important to assess the effect of breastfeeding on postpartum CVD risk in this cohort separately, to support specialised and ongoing breastfeeding support as part of postpartum lifestyle prevention of CVD.

Metabolic syndrome (MetS), a cluster of the most dangerous heart disease risk factors, is reduced in women who breastfeed for longer (13). It is also an appropriate marker of heart disease risk in women in early postpartum following a pregnancy complication (14). The primary aim of this study was to assess the effect of breastfeeding on the prevalence of MetS in early postpartum, amongst women with previous pregnancy complications who attended a postpartum cardiovascular preventative clinic in metropolitan South Australia. We also aimed to evaluate the frequency of breastfeeding initiation in this group.

## 2 Methods

### 2.1 Study design and setting

This is a cross-sectional analysis of data from women who attended a nurse-practitioner led postpartum cardiovascular disease prevention clinic at the Lyell McEwin Hospital (LMH), South Australia from August 2018 to December 2023. Clinical, anthropometric and questionnaire data from all clinic attendees were collected prospectively at the clinic as part of a purpose-built research registry linked to routine patient care. The LMH is a public tertiary-based hospital servicing the Northern Adelaide Local Health Network, providing adult cardiac and intensive care services, obstetric care and neonatal care for infants ≥32 weeks gestation. The NALHN catchment has the highest rates of coronary artery disease, diabetes, smoking and obesity in metropolitan Australia (15). This region is characterized as one of the most socioeconomically disadvantaged regions in metropolitan Australia (16).

### 2.2 Inclusion and exclusion criteria

The clinic, its premise and study procedures have been described previously. The research registry and associated study have been approved by the Central Adelaide Local Health Network (HREC/16/TQEH/256). The ethics committee waived the requirement for participant consent as the project's primary purpose is for quality assurance.

Briefly, women who had at least one of the following pregnancy complications, irrespective of age or parity, are referred for the clinic at the time of their baby's birth (Table 1). These complications of pregnancy are defined as per the South Australian Perinatal Practice Guidelines. Those with postpartum cardiomyopathy or pre-existing cardiovascular disease, those with only non-mediated GDM or HDP, or those with pre-pregnancy CVD risk factors that were not induced through pregnancy are not referred to the clinic (with the exception of those with pre-existing CVD risk factors developing a *de novo* complication e.g., T2DM developing HDP are still eligible for referral).

**Table 1:** Referral criteria for postpartum clinic.

Clinic referral criteria
Hypertensive disorders of pregnancy (including gestational hypertension, preeclampsia, eclampsia and HELLP syndrome), requiring medical therapy or resulting in <37 weeks gestation
Gestational diabetes mellitus requiring metformin or insulin therapy
Preterm delivery at <37 weeks gestation
Delivery of a small for gestational age infant
Placental abruption

Participants attend a postpartum appointment 6 months after birth of their baby. Demographic, medical, obstetric, breastfeeding history and dietary data were obtained from the participant by the clinic's medical scientist through self-report. Relevant medical and obstetric history including diagnosis of pregnancy complication/s were corroborated through medical notes. Socioeconomic status was calculated using a Socioeconomic Index For Area Index of Relative Socioeconomic Disadvantage (SEIFA IRSD) score. This index is a general measure of relative disadvantage of each local postcode, with a score >1,000 reflecting a lack of disadvantage and a score <1,000 indicating relative greater disadvantage.

Women were asked whether they breastfed at all during their index pregnancy, and how long or if they were still breastfeeding at the time of attending their appointment. Blood pressure measurements, including non-conventional measurements such as central blood pressure, were taken with an USCOM BP + device after the participant had been seated for 20 min and using the correct cuff size based on arm circumference. This device has been validated for use in pregnant women (17). Anthropometric measurements such as height, weight and waist circumference were measured. Women provided fasting blood samples to assess cardiovascular risk factors including markers of glycaemia, lipids and insulin. All results were discussed by an expert nurse practitioner who provided clinical guidance on cardiovascular risk, and advice on healthy lifestyle and diet.

The primary outcome of this study was metabolic syndrome (MetS), defined using the *Harmonising the Metabolic Syndrome* definition (18), which includes the presence of at least 3 of the 5 following risk factors:

Elevated waist circumference >80 cm (indicative of abdominal obesity across females of all ethnicities);
Elevated triglycerides >1.7 mmol/L; or drug treatment for elevated triglycerides
Reduced HDL-C < 1.3 mmol/L; or drug treatment for reduced High Density Lipoprotein Cholesterol) (HDL-C)
Elevated systolic blood pressure (SBP) >130 mmHg and or diastolic blood pressure (DBP) >85 mmHg; or use of antihypertensive medication in a woman with a history of hypertension
Elevated fasting glucose; or drug treatment of elevated glucose

Secondary outcomes were individual cardiovascular risk factors, including body mass index, waist circumference, peripheral and central blood pressure, triglycerides, HDL and Low Density Lipoprotein (LDL) cholesterol, total cholesterol, fasting plasma glucose and fasting insulin.

### 2.3 Assessment of breastfeeding

Women were considered to have initiated breastfeeding if they answered yes to “did you breastfeed?”. Breastfeeding exposure was defined as the reported number of consecutive months a woman had self-reported breastfeeding. If a woman reported still breastfeeding at the time of the appointment, the length of breastfeeding was recorded as the number of months postpartum the woman was when attending their appointment. Other sources of feeding (i.e., formula, solids), and reasons for not breastfeeding or discontinuation of breastfeeding were not routinely recorded at the time of analysis.

### 2.4 Statistical analysis

Statistical analysis was completed on IBM SPSS 27. Descriptive data on demographics were reported for the group as either mean (SD) or *n* = (%). The number of women who reported breastfeeding at any time point and the number of women still breastfeeding at their appointment was reported as *n* = (%).

Chi-square analysis was performed to assess the difference in the prevalence of MetS and individual components between those who reported breastfeeding for a duration of >5.5 months compared to those who did not, including those reported never initiating breastfeeding (0 months). Data reported as *n* = (%). A cut-off of 5.5 months was selected as there is variability in timing of the 6 month visit and many women were scheduled prior to 6 months postpartum [Mean 7.4 months (SD 2.1)]. To account for this further, we conducted a sensitivity analysis for women who attended the clinic between 5 and 7 months postpartum, excluding women who attended their appointment <5 months postpartum or >7 months postpartum. Student t-tests were performed to assess the difference individual cardiovascular risk factors between groups, with data reported as mean (SD).

Multivariate binary logistic regression was performed where breastfeeding for >5.5 months (yes/no) was the reported binary variable and the outcome was MetS. Data were reported as both unadjusted and adjusted odds ratio (adjusted for referral pregnancy complication, maternal age, and gravidity, socioeconomic status, smoking history and BMI at booking). These variables were selected ad-hoc, based on whether they influence both breastfeeding length of time and postpartum metabolic syndrome prevalence.

## 3 Results

### 3.1 Demographics

A total of 524 women were referred to and attended a clinic appointment from 7 August 2018 until 19 December 2023. The clinic reports a 56% attendance rate of those who are referred. Their demographics are reported in Table 2. The mean age of the group was 33.5 ± 6.7 years. Women attended their clinic appointment at 7.6 months (Range 17.6). The mean BMI was 31.3 ± 8.6 kg/m^2^, and the mean waist circumference was 98.8 ± 17.8 cm. 25.4% of participants were overweight, and 43.7% were obese based on BMI at their first antenatal appointment. Majority of women were Caucasian (56.3%). Most women had a Diploma or Trade Certificate (38.2%). A total of 62.9% of participants were married. The majority of referrals for the clinic were for GDM requiring metformin or insulin (62.2%), followed by medicated HDP (38.9%), SGA/IUGR (10.9%), sPTB (4.2%) and placental abruption (13.2%). 29% of the women had 2 or more pregnancy complications in their index pregnancy (including non-medicated complications like diet controlled GDM). A total of 13% of participants have had a previous maternal pregnancy complication prior to their index pregnancy. The mean parity in the group was 2.1.

**Table 2:** Maternal characteristics of clinic attendees.

Clinic attendees (*n* = 524)	Mean ± SD or *n* (%)
Ethnicity	
Caucasian	295 (56.3%)
Chinese and South East Asian	80 (15.3%)
Indian Subcontinent	55 (10.5%)
African	23 (4.4%)
Middle Eastern	44 (8.4%)
Hispanic	3 (0.6%)
Aboriginal	14 (2.7%)
BMI at booking (first antenatal appointment)	30.6 ± 8.4
BMI Catergories [*n* = (%)]	
Healthy (20–25 kg/m^2^)	114 (21.8%)
Overweight (25–29.9 kg/m^2^)	133 (25.4%)
Obese (>30 kg/m^2^)	229 (43.7%)
Underweight (<19 kg/m^2^)	4 (0.6%)
Referral Complication^a^	
GDM	326 (62.2%)
sPTB	22 (4.2%)
SGA/IUGR	57 (10.9%)
HDP	204 (38.9%)
PA	13 (2.5%)
History of pregnancy complication(s) prior to index pregnancy	69 (13.2%)
Reported 2 pregnancy complications	120 (22.9%)
Reported 3	25 (4.8%)
Reported 4	7 (1.3%)
Gravidity	2.8 ± 2.0
Months postpartum at appointment	7.6 ± 2.1
Age at appointment	33.5 ± 6.7
SEIFA IRSD	920.5 ± 70.7
Marital Status	
Married	329 (62.9%)
De-Facto	137 (26.1%)
Relationship, not living together	9 (1.7%)
Separated	5 (1.0%)
Single	44 (8.0%)
Educational Level^b^	
=<Year 9	17 (3.6%)
Year 10	27 (5.2%)
Year 11	32 (6.1%)
Year 12	69 (13.2%)
Diploma/Certificate	200 (38.2%)
Bachelor	110 (21%)
Higher Degree	39 (7.4%)
Current smoker	51 (9.8%)
History of smoking ever	97 (18.5%)
Minutes of Exercise Per Week	88.1 ± 115.8
BMI in early postpartum (Mean SD)	31.3 ± 8.6
BMI Catergories [*n* = (%)]	114 (18.1%)
Healthy (20–25 kg/m^2^)	134 (25.6%)
Overweight (25–29.9 kg/m^2^)	229 (43.7%)
Obese (>30 kg/m^2^)	4 (0.8%)
Underweight (<19 kg/m^2^)	
Waist Circumference	98.8 ± 17.8
T2DM (*n*=)(%)	22 (4.2%)
Hypertension (*n*=)(%)	59 (11.3%)
CKD (*n*=)(%)	13 (2.5%)

SD, standard deviation; SEIFA IRSD, Socioeconomic Index For Area Index of Relative Socioeconomic Disadvantage; BMI, body mass index; GDM, gestational diabetes mellitus; sPTB; SGA/IUGR, small for gestational age/intrauterine growth restriction; HDP, hypertensive disorder of pregnancy; PA, placental abruption.

^a^ Pregnancy complications are not mutually exclusive and one participant may be referred for ≥  1 complication of pregnancy. Pregnancy complications are reflective of those in the index pregnancy that were considered during the referral at the time of discharge from delivery of the infant/s.

^b^ Unknown for 23 participants.

### 3.2 Breastfeeding prevalence

A total of 445 (84.9%) women self-reported that they initiated breastfeeding. Of these women, 277 (62.2%) were currently breastfeeding at the time of their appointment. Overall, the mean breastfeeding length of the total group was 5.2 ± 3.0 months (Table 3).

**Table 3:** Clinic attendees who reported breastfeeding at postpartum follow-up and average length of breastfeeding duration.

Clinic attendees (*n* = 524)	*n* (%)
Initiated breastfeeding	445 (84.9%)
Currently breastfeeding	277 (62.4%)
Average length of breastfeeding for all attendees^a^	5.2 ± 3.0
Average length of breastfeeding in months for those who ceased breastfeeding before baseline appointment (*n* = 173)^a^	2.12 ± 1.41

^a^ Reported as Mean ± SD.

### 3.3 Prevalence of MetS and components

We could report metabolic syndrome status in 465 participants based on those who completed all cardiovascular assessments. The prevalence of MetS among those who breastfed for >5.5 months was 24.1%, compared to 60.5% for women who breastfeed for ≤5.5 months (Table 4). Those who breastfed for ≤5.5 months had a significantly higher prevalence of reduced HDL-cholesterol (55% vs. 45%), raised triglycerides (56.9% vs. 46.7%) and treated hypertension/current hypertension based on SBP and/or DBP (56.5% vs. 43.5%), SBP > 130mmHg (55.6% vs. 44.4%) compared to those who breastfed ≤5.5 months, respectively.

**Table 4:** Prevalence of MetS components and MetS amongst clinic attendees reporting breastfeeding > 5.5 months postpartum.

MetS components	n	Breastfeeding > 5.5 months (*n* = 265)	Breastfeeding ≤ 5.5 months (*n* = 231)	*P*-value
Months postpartum at appointment (Mean ± SD)	524	7.5 ± 2.3	7.5 ± 2.1	**-**
MetS	465^a^	64 (24.1%)	98 (60.5%)	**<0**.**001**
Abdominal obesity (Waist Circumference ≥80 cm)	491	222 (52%)	205 (48%)	0.687
Reduced HDL Cholesterol <1.29 mmol/L	478	98 (45%)	120 (55%)	**0**.**001**
Raised triglycerides ≥1.7 mmol/L	479	50 (43.1%)	66 (56.9%)	**0**.**014**
Raised fasting plasma glucose ≥ 5.6 mmol/L or treatment for T2DM	477	42 (46.7%)	48 (53.3%)	0.179
Treated hypertension, SBP ≥130 mmHg or DBP ≥85 mmHg	496	70 (43.5%)	91 (56.5%)	**0**.**012**
SBP ≥130 mmHg	493	59 (44.4%)	74 (55.6%)	**0**.**047**
DBP ≥85 mmHg	493	31 (43.1%)	41 (56.9%)	0.111

MetS and individual components are defined based on the Harmonising the metabolic syndrome definition. Data reported as *n* (%) unless otherwise stated. MetS, metabolic syndrome.

Bold indicates statistical significance as per *p*-value <0.05.

^a^ Data availability based on participant count that completed all components of cardiovascular assessment and fasting blood test.

**Table 5:** Association between breastfeeding and MetS or individual components based on multivariate logistic regression.

MetS components	Unadjusted	Adjusted^a^
Metabolic Syndrome (Yes v No)	**0.43** (**0.29–0.64)**	**0.47** (**0.29–0.77)**
Abdominal obesity (high v normal)	0.90 (0.53–1.5)	0.54 (0.22–1.3)
HDL (abnormal v normal)	**0.54** (**0.37–0.78)**	**0.55** (**0.35–0.87)**
Triglycerides (abnormal v normal)	**0.59** (**0.39–0.9)**	**0.53** (**0.31–0.89)**
Glucose (abnormal v normal)	0.73 (0.47–1.2)	0.7 (0.41–1.3)
SBP (abnormal v normal)	0.67 (0.40–1.1)	0.61 (0.32–1.1)
DBP (abnormal v normal)	**0.67** (**0.4–0.9)**	0.82 (0.49–1.4)

Bold indicates statistical significance as per *p*-value <0.05.

^a^ Adjusted for SEIFA IRSD, Age, Gravidity, Booking BMI, history of smoking (Yes/No), Hypertensive Disorders of pregnancy, Gestational diabetes mellitus, Small for Gestational Age Intrauterine Growth Restriction, spontaneous Preterm Birth and Placental Abruption.

Supplementary Table S1 demonstrates the individual cardiometabolic risk factors amongst women who reported breastfeeding for >5.5 months compared to those who breastfed for ≤5.5 months. Women who breastfed for > 5.5 months had significantly lower mean BMI (30.6 ± 7.6 kg/m^2^ vs. 32.0 ± 9.6 kg/m^2^, *p* = 0.003) and mean waist circumference (96.7 ± 15.5 cm vs. 100.6 ± 19.7 cm, *p* = 0.002) than women who breastfed for ≤5.5 months, respectively. Women who breastfed for > 5.5 months had significantly lower mean peripheral DBP (73.4 ± 9.1 mmHg vs. 74.7 ± 11.4 mmHg, *p* = 0.010) and mean central DBP (75.8 ± 9.2 mmHg vs. 76.9 ± 11.6 mmHg, *p* = 0.003) compared to women who breastfed for ≤5.5 months, respectively.

### 3.4 The association between breastfeeding length and MetS in postpartum

The effect of breastfeeding for >5.5 months on the odds of having MetS and its components is reported in Table 5. Women who breastfed for >5.5 months were 53% less likely to have MetS at 6 months postpartum than women who did not breastfeed for >5.5 months (aOR 0.47; 95% CI 0.29–0.77). Further, women who breastfed for a duration of > 5.5 months were 45% less likely to have reduced HDL <1.29 mmol/L (aOR 0.55; 95% CI 0.35–0.87) and 47% less likely to have raised triglycerides ≥1.7 mmol/L (aOR 0.53; 95% CI 0.31–0.89) at 6 months postpartum (Table 5). These findings were adjusted for covariates such as individual pregnancy complications, smoking history, early pregnancy BMI and socioeconomic status, highlighting the association between breastfeeding length and MetS prevalence in early postpartum is independent of these factors.

### 3.5 Breastfeeding length stratified by referral pregnancy complication

Table 6 demonstrates the comparison of referral pregnancy complications between women who breastfed for >5.5 months and women who breastfed for ≤5.5 months. Participants referred to the clinic for GDM more commonly reported that they breastfed for a duration of >5.5 months compared to those that were not (54.5% vs. 44.4%, *p* < 0.001). Conversely, participants referred to the clinic for HDP less frequently reported breastfeeding for a duration of >5.5 months than participants referred for non-HDP (40.9% vs. 59.1%, *p* < 0.001).

**Table 6:** Comparison of the proportion of referrals based on pregnancy complications between participants who breastfed for a duration of >5.5 months and those who did not^a^.

Complication	Breastfeeding > 5.5 months (*n* = 265)	Breastfeeding ≤ 5.5 months (*n* = 231)	*P*-value
HDP	84 (40.9%)	120 (59.1%)	**<0**.**001**
GDM	180 (55.6%)	144 (44.4%)	**0**.**025**
SGA/IUGR	26 (45.6%)	31 (54.4%)	0.327
sPTB	12 (54.5%)	10 (45.5%)	0.787
PA	6 (50%)	6 (50%)	0.903

SD, standard deviation; SEIFA IRSD, Socioeconomic Index For Area Index of Relative Socioeconomic Disadvantage; BMI, body mass index; GDM, gestational diabetes mellitus; sPTB,; spontaneous preterm birth; SGA/IUGR, small for gestational age/intrauterine growth restriction; HDP, hypertensive disorder of pregnancy; PA, placental abruption.

Bold indicates statistical significance as per *p*-value <0.05.

^a^ Pregnancy complications are not mutually exclusive and one participant may be referred for >1 complication of pregnancy. Pregnancy complications are reflective of those in the index pregnancy that were considered during the referral at the time of discharge from delivery of the infant/s.

### 3.6 Sensitivity analysis

Sensitivity analysis was conducted for participants who attended their appointment between 5 and 7 months postpartum (*n* = 273) (Supplementary Tables S2 and S3). The analysis showed that breastfeeding for > 5.5 months was associated with lower serum triglycerides, and there was no difference between groups for mean peripheral and central diastolic blood pressure (Supplementary Table S2). However, there was a greater proportion of women who did not breastfeed for >5.5 months with a diastolic blood pressure ≥85 mmHg compared to those who did breastfeed for >5.5 months (Supplementary Table S3). All other results were similar between the primary and sensitivity analyses.

We looked at differences between those who did and did not initiate breastfeeding. The prevalence of MetS was higher in women who never initiated breastfeeding (52.6% vs. 28.3%, *p* < 0.001). We found that those who did not initiate breastfeeding had significantly higher BMI, waist circumference, peripheral systolic and diastolic blood pressure, higher central systolic blood pressure, significantly higher triglycerides, LDL-C than those who did initiate breastfeeding (all *p* < 0.05). Furthermore, HDL-C was significantly lower in those who never initiated breastfeeding (Supplementary Table S4).

## 4 Discussion

### 4.1 Summary of findings

The aim of this cross-sectional study was to evaluate the association between breastfeeding duration and the prevalence of MetS amongst women with previous pregnancy complications. Our analysis revealed that women who breastfed for >5.5 months were less likely to have MetS and fewer CVD risk factors at 6 months postpartum, independent of known risk factors for CVD. Our findings suggest that breastfeeding duration is associated with more favourable postpartum cardiometabolic health. Our findings highlight the importance of supporting breastfeeding in women with prior pregnancy complications as part of postpartum cardiometabolic disease risk management.

Being overweight/obese is the most common risk factor for MetS and contributes significantly to the development of the other MetS components. Interestingly, while our descriptive analysis revealed that women who breastfed for > 5.5 months had a lower mean BMI and waist circumference than those who breastfed ≤5.5 months, the prevalence of abdominal obesity as a MetS component was not different between groups. This is likely due to the overall cross-sectional sample being overweight and obese. Breastfeeding has high caloric expenditure, and reducing pregnancy weight in the postpartum period reduces the prevalence of long-term obesity and chronic disease. However, women who are already obese in pregnancy are more likely to have delayed lactogenesis (19). Therefore, weight management should encourage pre-conception to optimise breastfeeding outcomes, which will improve postpartum weight reduction and subsequent prevalence of other CVD risk factors.

We found that women who breastfed for >5.5 months postpartum had reduced dyslipidaemia compared to their counterparts. Our results are in line with those of Yu et al. (2020) and their cross-sectional analysis of a postpartum health clinic, identifying a dose-response effect of increased breastfeeding on lipid markers (20). A cohort study by Niu et al. (2022) found that breastfeeding for 12 months postpartum was associated with a reduced atherogenic profile compared to not breastfeeding (21). Breastfeeding promotes lipolysis and mobilisation of lipids from non-adipose tissue into breastmilk, resulting in a reduction in triglycerides and VLDL-C, and a subsequent increase in serum HDL-C levels (22, 23). Breastfeeding is likely to improve the postpartum lipid profile and may be a mechanism to reduce long-term atherosclerosis in women with previous pregnancy complications.

Women who breastfed for ≤5.5 months had a higher prevalence of SBP >130 mmHg and use of antihypertensive medications at baseline. A meta-analysis of risk showed that breastfeeding for >12 months reduces hypertension risk by 13% (8). It is postulated that release of oxytocin and prolactin as a neuroendocrine response to lactation mediates blood pressure and subsequent hypertension risk; however, studies looking at the immediate effects of breastfeeding on postpartum blood pressure are still limited (24, 25). Previous research from our group has shown that women who experienced a major pregnancy complication and reported breastfeeding (at any intensity) for at least 6 months had improved peripheral and central blood pressure, and improved mean arterial blood pressure compared to those who did not breastfeed at 3 years postpartum (26). Interestingly, when we adjusted for known covariates including individual HDPs, smoking history, early pregnancy BMI and socioeconomic status the association between breastfeeding status and blood pressure variables remained. This suggests that the association between breastfeeding length and vascular health is independent of these established hypertensive risk factors. Overall, women who breastfeed for 6 months or longer are less likely to have impaired vascular health than those who breastfeed for a shorter duration.

The percentage of women who reported breastfeeding for any length of time was 85%, with 15% reporting that they never initiated breastfeeding. This rate is lower than the national average rate of 96% mothers initiating breastfeeding (27). Patients in this study are representative of women with severe pregnancy complications, higher rates of obesity and socioeconomic disadvantage. These factors are all associated with an inability to breastfeed and early cessation of breastfeeding (17, 28, 29). A cross-sectional online survey of Australian women diagnosed with GDM found that poor home support, obesity, low income, socioeconomic disadvantage and prenatal smoking were some factors associated with breastfeeding cessation (30). Further, 15% of our patients were referred for spontaneous preterm birth or SGA alone, not accounting for those who were referred for HDP. Women who have these complications may have delayed lactogenesis due to be separated while their infants are in Neonatal Intensive Care Unit (NICU). A retrospective analysis of pregnant women with premature pre-labour rupture of membranes identified that only 86% of women initiated breastfeeding prior to discharge and only 73% were still breastfeeding at 6 weeks (31). Our analysis reveals a group of women who may benefit from greater lactation support, with greater efforts needed to understand the practical, psychosocial and physiological barriers to breastfeeding.

We identified that 60% of women referred for HDP did not breastfeed for >5.5 months. Horsely et al. (2022), in a prospective cohort analysis, found that women who had HDP breastfed for a shorter duration and were more likely to be non-exclusively breastfeeding at 4 months postpartum compared to their counterparts (32). Prior research suggests that women with previous HDP may have difficulty with milk production due to physiological impacts from obesity (33). Interestingly, we found that 56% of women who were referred for GDM breastfed for >5.5 months compared to 44% who did not. These results are surprising because women with GDM are more likely to have delayed lactogenesis and cessation of breastfeeding that non-GDM women (29), likely influenced by obesity. However, in our group, mean BMI was not significantly different between those referred for HDP compared to GDM (data not shown). While Aldridge et al. (2023) found that the knowledge of CVD risk after a pregnancy complication was similar between HDP and GDM groups in our groups (34), women with GDM are provided with greater support and information regarding T2DM risk during their diabetes education at our centre (or similar). This support includes being encouraged to breastfeed exclusively for at least 6 months. Having this knowledge regarding breastfeeding benefits, together with offering optimal dietary and medication support during pregnancy, may promote greater rates of breastfeeding initiation and longer duration. This finding may be reflective of knowledge of breastfeeding benefits in our cross-sectional group. Qualitative research is needed to determine the knowledge and thoughts of women with previous pregnancy complications regarding breastfeeding and future CVD health.

### 4.2 Limitations

There are a number of strengths of this study. We analysed a unique cross-sectional group of women at high risk for future cardiovascular disease due to their pre-existing pregnancy complications and low socioeconomic status. We have analysed data for women with other placental conditions such as sPTB, SGA and placental abruption which are less represented in the literature on major pregnancy complications and maternal CVD risk. Our analysis exclusively included women from an outpatient clinic which reduces self-selection bias. We also used MetS as a surrogate marker of cardiovascular risk, which is more suitable for a young population than traditional CVD risk factors.

There are several limitations that warrant discussion. First, majority of women were referred for HDP and GDM. The minority of referred women had a purely placental complication of pregnancy (i.e., spontaneous preterm birth, stand-alone IUGR and placental abruption). These complications individually are associated with an increased risk of cardiovascular disease, and it is well established women with these complications face significant physical barriers to breastfeeding like infant separation. Therefore, future studies should assess cardiometabolic health individually in this group without influence of hypertensive disorders to elucidate how breastfeeding length can improve cardiometabolic risk in this high-risk group.

The participants self-reported their length of breastfeeding, meaning our analysis is subject to recall bias. However, a systematic review of maternal recall validity regarding breastfeeding practices by Li et al. (2005) found that in mothers with a recall period of <3 years, the majority of women accurately recalled duration of breastfeeding within a one month period (19). Further, we did not collect information on the extent of lactation intensity, whether current breastfeeding status was exclusive or mixed, or information on why women stopped breastfeeding. A major limitation of the current literature is the variation of how breastfeeding is defined and data is collected (i.e., prospectively or retrospectively), leading to inconsistences in the evidence that cannot be translated into clinical guidelines. Future studies should look at lactation across a spectrum to determine how the intensity of lactation is associated with cardiometabolic outcomes in women. This data is now routinely collected in our postpartum cardiovascular disease prevention clinic in order to facilitate future analyses of breastfeeding intensity.

Furthermore, for women who ceased breastfeeding before 6 months postpartum or never initiated breastfeeding we do not have data on the reasons for why this is in our patients, including whether this was due to patient-reported stressors or medically indicated. For women who were on antihypertensive medications at the time of their appointment, only 30% of them reported never breastfeeding (data not shown) although majority of women were on medications deemed breastfeeding safe in Australia. Furthermore, as women are only seen initially at 6 months postpartum we know very minimal about how their primary physician managed certain medical conditions and treatment, which may have influenced breastfeeding cessation. We are now collecting information on patient-reported reasons for breastfeeding cessation to further understand barriers to breastfeeding continuation in this cohort.

Unfortunately, there is no data on whether participant's had pre-existing metabolic syndrome or cardiovascular risk factors prior to pregnancy, including pre-pregnancy anthropometrics. Therefore, we cannot infer causality. Given the socioeconomic disadvantage of the population, and the mean BMI of the group at their booking visit (generally <20 weeks gestation), it is likely that many of the participants had pre-existing cardiometabolic risk factors (particularly obesity) prior to pregnancy which would influence lactation, pregnancy complication development and subsequent postpartum metabolic syndrome status. While adjusting for BMI at booking did not influence the association between breastfeeding and MetS, future studies require assessment of pre-pregnancy health status to validate our findings.

## 5 Conclusion

Women who breastfeed for a longer duration were associated with having more favourable cardiometabolic outcomes at 6 months postpartum, including a lower prevalence of MetS in early postpartum. These findings highlight the potential role of breastfeeding as an important factor in postpartum cardiometabolic health after a pregnancy complication. Future research should assess pre-pregnancy cardiometabolic health, lactation intensity and the risk of metabolic syndrome, to provide a better understanding on whether there is an independent relationship between breastfeeding and cardiovascular disease risk that can be translated into clinical practice.

## Data Availability

The raw data supporting the conclusions of this article will be made available by the authors upon reasonable request, without undue reservation.
