# Supplementary material for: The association between breastfeeding and prevalence of metabolic syndrome in women with a previous major pregnancy complication
Source: Front Glob Womens Health. 2026 Feb 24;7:1625603. doi: 10.3389/fgwh.2026.1625603 (PMC12971975; doi:10.3389/fgwh.2026.1625603)
Supplement: Supplementary file 1 [file Supplementaryfile1.docx]

Supplementary Material

***Supplementary Table 1: Cardiovascular and metabolic risk factors among clinic attendees who did and did not report breastfeeding ≥ 5.5 months postpartum at their appointment***

|  | **Breastfeeding ≥ 5.5 months (n=265)** | **Breastfeeding < 5.5 months  (n=231)** | **P-value** |
| --- | --- | --- | --- |
| BMI | 30.6 (7.6) | 32.0 (9.6) | **0.003** |
| Waist Circumference | 96.7 (15.7) | 100.6 (19.7) | **0.002** |
| Peripheral SBP* | 121.1 (12.9) | 124.0 (14.1) | 0.34 |
| Peripheral DBP* | 73.4 (9.1) | 74.7 (11.4) | **0.010** |
| Central SBP* | 114.2 (14.5) | 117.0 (14.6) | 0.70 |
| Central DBP | 75.8 (9.2) | 76.9 (11.6) | **0.003** |
| Triglycerides | 1.23 (1.1) | 1.49 (0.9) | 0.44 |
| HDL Cholesterol | 1.43 (1.0) | 1.27 (0.3) | 0.21 |
| Serum Glucose | 5.2 (2.9) | 5.3 (1.5) | 0.28 |
| Insulin | 14.6 (17.2) | 16.3 (14.2) | 0.96 |

^Data Reported as Mean ± SD^

***Supplementary Table 2: Sensitivity analysis - Cardiovascular and metabolic risk factors among COFFEE attendees who did and did not report breastfeeding > 5.5 months postpartum at baseline seen between 5-7 months postpartum***

|  | **Breastfeeding ≥ 5.5 months (n=146)** | **Breastfeeding < 5.5 months  (n=127)** | **P-value** |
| --- | --- | --- | --- |
| BMI | 29.5 (7.4) | 31.4 (9.5) | **0.021** |
| Waist Circumference | 94.8 (14.5) | 101.2 (19.7) | **0.003** |
| Peripheral SBP* | 119.5 (12.6) | 123.4 (14.2) | 0.365 |
| Peripheral DBP* | 72.6 (9.8) | 73.5 (10.7) | 0.887 |
| Central SBP* | 112.5 (15.3) | 115.9 (14.5) | 0.799 |
| Central DBP | 74.7 (9.7) | 75.7 (11.0) | 0.573 |
| Triglycerides | 1.1 (0.7) | 1.5 (1.3) | <0.01 |
| HDL Cholesterol | 1.5 (1.3) | 1.3 (0.4) | 0.184 |
| Serum Glucose | 5.1 (1.3) | 5.2 (1.4) | 0.974 |
| Insulin | 13.7 (19.1) | 16.2 (15.7) | 0.634 |

^Data Reported as Mean ± SD^

***Supplementary Table 3: Sensitivity analysis - Prevalence of MetS components and MetS amongst COFFEE attendees who did and did not report breastfeeding > 5.5 months postpartum at baseline seen between 5-7 months postpartum***

|  | **Breastfeeding ≥ 5.5 months (n=146)** | **Breastfeeding < 5.5 months  (n=127)** | **P-value** |
| --- | --- | --- | --- |
| Abdominal obesity (Waist Circumference ≥80cm) | 122 (83.6%) | 111 (88.8%) | 0.216 |
| Reduced HDL Cholesterol <1.29mmol/L | 50 (35%) | 59 (51%) | **0.010** |
| Raised triglycerides ≥1.7mmol/L | 23 (16.1%) | 37 (31.9%) | **0.003** |
| Raised fasting plasma glucose ≥5.6mmol/L or treatment for T2DM | 21 (14.8%) | 23 (19.7%) | 0.299 |
| Treated hypertension, SBP ≥130mmHg or DBP ≥85mmHg | 33 (22.8%) | 45 (35.2%) | **0.024** |
| SBP ≥130mmHg | 18 (12.4%) | 14 (10.9%) | 0.705 |
| DBP ≥85mmHg | 27 (18.6%) | 37 (28.9%) | **0.045** |
| MetS* | 30 (21.6%) | 48 (41%) | **<0.001** |

^MetS and individual components are defined based on the Harmonising the metabolic syndrome definition.
*Data reported as n= (%). MetS n=256 due to reduced compliance with blood test^

***Supplementary Table 4: Sensitivity analysis - Cardiovascular and metabolic risk factors among COFFEE attendees who reported not initiating breastfeeding compared to initiating breastfeeding***

|  | **Breastfeeding ≥ 5.5 months (n=445)** | **Breastfeeding < 5.5 months  (n=75)** | **P-value** |
| --- | --- | --- | --- |
| BMI | 30.6 (8.3) | 34.5 (8.8) | **<0.001** |
| Waist Circumference | 97.8 (17.3) | 106.1 (19.0) | **<0.001** |
| Peripheral SBP* | 112.1 (22.2) | 122.3 (19.1) | **<0.001** |
| Peripheral DBP* | 83 (20.8) | 78.5 (15.1) | **0.039** |
| Central SBP* | 107.2 (20.2) | 115 (17.8) | **0.01** |
| Central DBP | 76.1 (10.7) | 77.90 (10.7) | 0.09 |
| Total Cholesterol | 4.7 (2.5) | 5.3 (4.6) | **0.06** |
| Triglycerides | 1.3 (1.1) | 1.6 (1.1) | **0.01** |
| HDL Cholesterol | 1.3 (0.7) | 1.2 (0.2) | **0.02** |
| LDL Cholesterol | 2.7 (0.7) | 3.2 (4.3) | **0.009** |
| Serum Glucose | 5.2 (2.4) | 5.2 (0.9) | 0.942 |
| Insulin | 14.8 (14.9) | 17.9 (13.7) | 0.110 |
